# Development and Evaluation of Novel Encapsulated Isoeugenol-Liposomal Gel Carrier System for Methicillin-Resistant *Staphylococcus aureus*

**DOI:** 10.3390/gels9030228

**Published:** 2023-03-15

**Authors:** Sulaiman Mohammed Alnasser, Faizul Azam, Mohammed H. Alqarni, Alhussain H. Aodah, Sana Hashmi, Mehnaz Kamal, Alotaibi Meshal, Aftab Alam

**Affiliations:** 1 Department of Pharmacology and Toxicology, Unaizah College of Pharmacy, Qassim University, Unaizah 51911, Saudi Arabia; 2 Department of Pharmaceutical Chemistry and Pharmacognosy, Unaizah College of Pharmacy, Qassim University, Unaizah 51911, Saudi Arabia; 3 Department of Pharmacognosy, College of Pharmacy, Prince Sattam Bin Abdulaziz University, Al Kharj 11942, Saudi Arabia; 4 Department of Pharmaceutics, College of Pharmacy, Prince Sattam Bin Abdulaziz University, Al Kharj 11942, Saudi Arabia; 5 Department of Pharmaceutical Sciences, Unaizah College of Pharmacy, Qassim University, Unaizah 51911, Saudi Arabia; 6 Department of Pharmaceutical Chemistry, College of Pharmacy, Prince Sattam Bin Abdulaziz University, Al Kharj 11942, Saudi Arabia; 7 Department of Pharmacy Practice, College of Pharmacy, University of Hafr Al Batin, Hafr Al-Batin 39911, Saudi Arabia

**Keywords:** antimicrobial, antibiotics, *Staphylococcus aureus*, MRSA, essential oils, isoeugenol

## Abstract

In recent years, methicillin-resistant *Staphylococcus aureus* (MRSA) bacteria have seriously threatened the health and safety of the world’s population. This challenge demands the development of alternative therapies based on plant origin. This molecular docking study ascertained the orientation and intermolecular interactions of isoeugenol within penicillin-binding protein 2a. In this present work, isoeugenol as an anti-MRSA therapy was selected by encapsulating it into a liposomal carrier system. After encapsulation into the liposomal carrier, it was evaluated for encapsulation efficiency (%), particle size, zeta potential, and morphology. The percentage entrapment efficiency (% EE) was observed to be 57.8 ± 2.89% with a particle size of 143.31 ± 7.165 nm, a zeta potential of (−)25 mV, and morphology was found to be spherical and smooth. After this evaluation, it was incorporated into a 0.5% Carbopol gel for a smooth and uniform distribution on the skin. Notably, the isoeugenol-liposomal gel was smooth on the surface with a pH of 6.4, suitable viscosity, and spreadability. Interestingly, the developed isoeugenol-liposomal gel was safe for human use, with more than 80% cell viability. The in vitro drug release study shows promising results with 75.95 ± 3.79% of drug release after 24 h. The minimum inhibitory concentration (MIC) was 8.236 µg/mL. Based on this, it can be concluded that encapsulating isoeugenol into the liposomal gel is a potential carrier for MRSA treatment.

## 1. Introduction

Increasing concerns about antibiotic resistance pose public health threats under current circumstances [1]. *Staphylococcus aureus* (*S. aureus*) is one of the most consequential human pathogens that has developed alarming resistance to various antibiotics in clinical practice [2]. However, it is very challenging for scientists and researchers to eliminate *S. aureus* infections, mainly due to biofilm development and drug resistance against several available antibiotics [3]. Despite this, there is a growing problem with the abuse and misuse of antibiotics, which is posing a threat to human health.

Methicillin-resistant *Staphylococcus aureus* (MRSA) is one of the most prevalent pathogens responsible for nosocomial infections in public health settings [4]. MRSA is one of the most frequently detected bacteria in hospitals worldwide, with several morbidity and mortality percentages [5]. It further increases hospital stays, unnecessarily increasing the government’s and society’s healthcare burden [6]. The World Health Organization (WHO) estimated that the chances of survival are low in the case of MRSA patients. It was estimated that 64% of these patients are more likely to die than non-MRSA patients [7]. Apart from MRSA being a leading source of morbidity and mortality on a global scale, it is majorly responsible for the high prevalence of skin infections [8]. The inability of conventional medicine to treat all diseases caused by superbugs is already a significant challenge [9]. The increasing number of infections caused by MRSA requires the development of a novel antibacterial agent to combat the formation of biofilms [10].

With the rapid rise in the number of cases of multi-resistant bacteria, there is a need to develop new ways to control the spread of resistant pathogens [11]. There has been an increase in the number of bacteria resistant to several antibiotics, which needs to find new strategies to block the spread of resistant pathogen types. Many plants in nature offer a variety of medicinal properties and a rich heritage of safety applications [12]. Based on this, researchers are looking for alternative therapeutics to treat MRSA.

Among them, an interesting topic is the use of essential oils (EO), which have antibacterial and antifungal effects and have been used in conventional therapy for centuries [13]. One of these natural substances is eugenol. Eugenol is the main ingredient extracted from the leaves and buds of *Eugenia cariophyllata* (clove), which belongs to the family Myrtaceae [14]. It has been demonstrated that eugenol EOs may be used in a wide range of sectors, including agriculture, flavorings, food, cosmetics, and medicines. Eugenol is widely known for having pharmacological effects, such as analgesics, bactericidal, chemotherapeutic, analgesic, antioxidants, and anti-inflammatory effects. Eugenol derivatives’ local anesthetic and antibacterial properties are well reported [15]. It has been shown time and again that the antibacterial properties of the isomer isoeugenol are better than those of its parent compound [16]. Compared to eugenol, isoeugenol is more effective and less genotoxic when treating MRSA. It also has a known antibacterial mechanism of action [17].

It is well known that EO has antibacterial properties; nevertheless, their use by healthcare professionals is limited. This is mainly because they are volatile, have low solubility in water, and are unstable when exposed to heat, light, and oxygen [18]. Encapsulating EOs in a variety of carrier systems, such as solid lipid nanoparticles [19], micelles [20,21], liposomes [22], cyclodextrins [23], and others too [18], are one of the most effective methods for overcoming the challenges presented by these issues. Although there have been many attempts to solve the problems of poor water solubility and instability, encapsulation in a liposomal gel is probably one of the best methods.

In treating MRSA infections, transdermal or topical administration is preferable and more helpful than other routes of administration, such as oral or intravenous administration. Unfortunately, drug delivery by transdermal administration is not easy due to the stratum corneum layer in the skin [24]. Liposomes are the most prevalent and extensively researched nanocarriers for targeted medication delivery via topical [25]. Nanoparticles such as liposomes, composed of phospholipids and correspond to the lipid bilayer of the cell membrane, are used to localize drugs in skin diseases and improve the bioavailability of these treatments in vivo [26]. A typical problem with liquid liposomal formulations is that the drugs may leak. In previous research, liposomes have been incorporated into the gel matrix to improve encapsulation stability with simple topical application [27].

Many studies on nano vesicular agents have gained tremendous popularity in recent decades as potential topical and transdermal delivery vehicles. Varona et al. encapsulated the essential oil of lavandin in liposomes using a thin film method. The average diameter of the developed formulation was 0.4–1.3 µm, and the entrapment efficiency was up to 66% [28]. Wolfram et al. developed hesperetin liposomal with the thin-layer evaporation technique. In his study, they reported no impairment of therapeutic efficacy. It showed good anticancer activity against H441 lung cancer cells and MDA-MB231 breast cancer cells. The long-term stability of liposomes was also seen in serum and storage conditions [29].

Thus, this study aims to explore the antimicrobial properties of isoeugenol using computational study and further preparation of a nano liposomal form of isoeugenol that can be applied topically and has the most significant antibacterial activity. Based on this hypothesis, firstly, the liposomes of isoeugenol were prepared using soya lecithin and cholesterol by the freeze–thaw method and further evaluated. After this, isoeugenol was incorporated with liposomes into a Carbopol 940 gel base to avoid leakage and increase stability. Furthermore, the developed isoeugenol-loaded liposomal gel was evaluated for rheological parameters, in vitro drug release, antibacterial, and stability studies. In addition, the intermolecular interaction of isoeugenol with penicillin-binding protein 2a was studied by molecular docking, followed by molecular dynamics simulation to ascertain the conformational stability of the docked complex in the living system.

## 2. Results and Discussion

### 2.1. Molecular Docking Study of Isoeugenol with Penicillin-Binding Protein

Rapid advancements in computer hardware and software have benefited the drug design and discovery field greatly, paving the way for new drug development at a substantially reduced time and cost. Therefore, molecular docking was implemented to understand the modus of binding interactions of isoeugenol with penicillin-binding protein 2a (PBP2a). Penicillin-binding proteins catalyze the last steps of the biosynthesis of peptidoglycan cell walls. PBP2a subunit mutations can reduce the effectiveness of β-lactam antibiotics, encouraging Staphylococcus aureus to grow without restriction and developing resistance. A heterocyclic antibiotic without a β-lactam ring, (E)-3-(2-(4-cyanostyryl)-4-oxoquinazolin-3(4H)-yl) benzoic acid, interacts non-covalently with the PBP2a allosteric site and suppresses PBP enzymatic activity in the presence of a double mutation in PBP2a (i.e., N146K and E150K) [30]. Therefore, the binding site of the quinazoline derivative was considered for the molecular docking of isoeugenol in this study.

**Figure 1:**
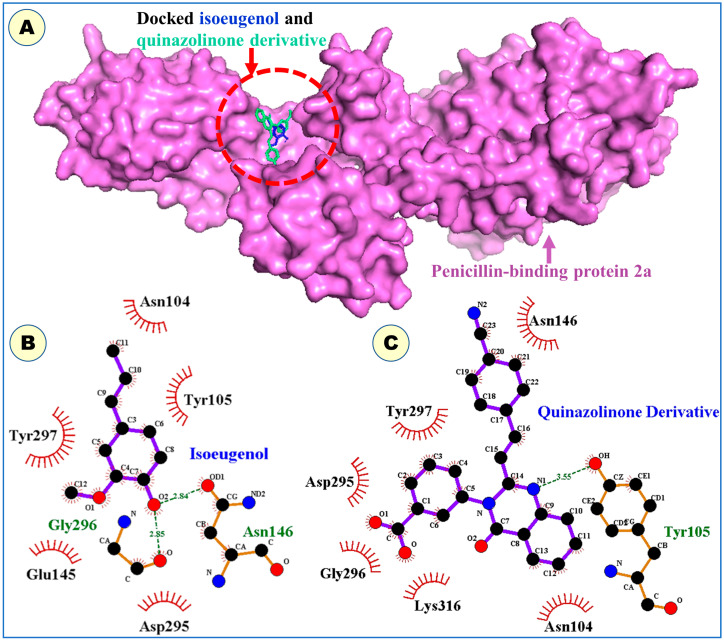
(**A**) Docking predicted molecular conformations of isoeugenol (shown as sticks in blue color) and quinazolinone antibiotic (depicted as sticks in cyan color) in the allosteric site of penicillin-binding protein 2a (shown as surface in magenta color). (**B**) Ligplot diagram of the docked isoeugenol showing binding residues. (**C**) Intermolecular interactions of quinazolinone antibiotic are shown by ligplot diagram.

The docking results revealed that isoeugenol binds to the allosteric site of PBP2a in a similar fashion as a native co-crystallized quinazolinone antibiotic, exhibiting a root-mean-square deviation of 1.55 Å (Figure 1). The binding energy of the best conformation of docked isoeugenol was recorded as −4.5 kcal/mol. The allosteric site of PBP 2a is surrounded by amino acid residues, such as Asn104, Tyr105, Asn146, Lys273, Glu294, Asp295, Gly296, Tyr297, Ile309, and Lys316 [30]. The docked compound occupies the binding site through two hydrogen bonds each with Asn146 and Gly296. Residues such as Asn104, Tyr105, Glu145, Asp295, and Tyr297 supported the isoeugenol molecule, aiding in non-polar connections within the allosteric site of PBP2a. However, the quinazolinone antibiotic participates in hydrogen bonding with Tyr105, whereas the rest of the residues support van der Waal’s interactions. These findings may be beneficial for novel drug development targeting penicillin-binding protein 2a of MRSA.

### 2.2. Molecular Dynamics Simulation

**Figure 2:**
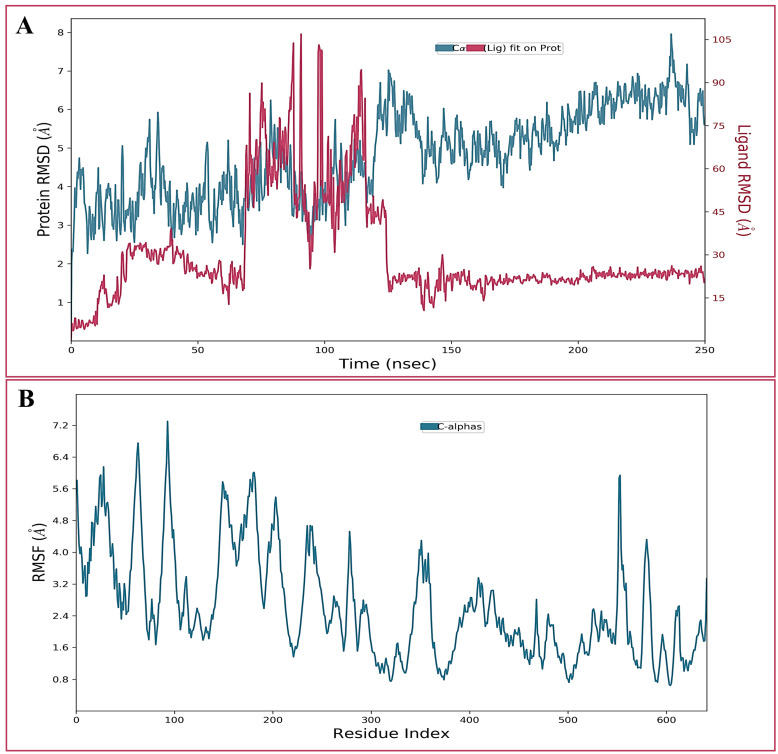
(**A**) The root-mean-square deviations (RMSD) of Cα atoms of penicillin-binding protein 2a in complex with isoeugenol during 250 ns MD simulation. (**B**) The root-mean-square fluctuation (RMSF) of Cα atoms of penicillin-binding protein 2a in complex with isoeugenol during 250 ns MD simulation.

Molecular dynamics (MD) simulation, a prevalent computer-assisted drug design approach, may be used to predict the kinetic and thermodynamic characteristics of biological systems under specified physiological conditions [31,32,33]. Therefore, the best-docked conformation of isoeugenol in complex with penicillin-binding protein 2a was subjected to MD simulation analysis to explore the stability of the ligand-protein complex as well as the principal intermolecular interactions along the simulated trajectory. Various conventional simulation parameters, such as backbone RMSDs for alpha-carbon atoms, were evaluated on the trajectories of the simulated complex. In addition, the root-mean-square fluctuations (RMSFs) of certain amino acid residues and intermolecular interactions were assessed. Figure 2A illustrates the RMSD curve for the simulated complex. As exhibited in the RMSD plot shown on the left side, the simulated system has equilibrated very well and the average fluctuations in the Cα atoms were noted as 4.79 Å. Nonetheless, small fluctuations are to be anticipated within the first 125 ns, after which the simulation trajectory stabilizes. Similarly, the RMSD of the bound ligand, seen on the right side of the plot, fluctuates during the initial 125 ns period before settling into a steady state for the remainder of the simulation trajectory.

The local conformational alterations along the penicillin-binding protein 2a chain were investigated by analyzing the RMSF during simulation time. As depicted in Figure 2B, loop regions usually fluctuate the most during simulation, though alpha-helices and beta-sheets were rigid.

**Figure 3:**
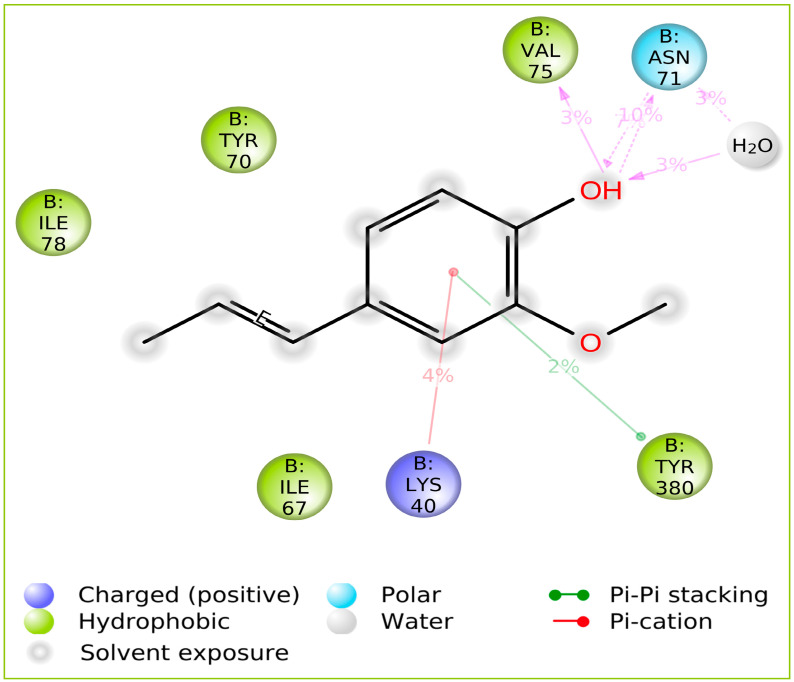
Isoeugenol in complex with penicillin-binding protein 2a of MRSA during MD simulation trajectories of 250 ns.

Simulation interaction diagrams presented in Figure 3 during the entire simulation time signify a comprehensive intermolecular interaction profile of isoeugenol with penicillin-binding protein. The modus of the interaction pattern of isoeugenol clearly illustrates that the docking-predicted main contacts were not preserved during the MD simulation. Tyr380 afforded π-π stacking interaction with the aromatic ring of the isoeugenol, while π-cation interaction was observed with Lys40 residue. Other hydrophobic residues include Tyr70, Val75, and Ile78. In addition, Asn71 contributed to the polar interaction with the phenolic hydroxyl group of isoeugenol.

### 2.3. % Entrapment Efficiency (% EE)

This present study developed a liposomal nanocarrier containing isoeugenol by combining soy phosphatidylcholine with cholesterol. It is effective, safe, non-toxic, and excellent for encapsulating essential oils such as isoeugenol. The % EE was determined using the UV-Vis spectrophotometry technique at 283 nm. The % EE was observed to be 57.8 ± 2.89%. Another researcher observed a % EE value by encapsulating *Heracleum persicum* L. essential oil in lecithin/cholesterol liposomes. Their study reported the % EE in the range of 51.93–68.13% [34]. The % EE of isoeugenol in liposomal nanocarrier will affect the nanoparticle’s size distribution and average particle size. It can be seen from the dynamic light scattering (DLS) and particle distribution index (PDI) study.

### 2.4. Determination of Particle Size, Size Distribution and Zeta Potential

**Figure 4:**
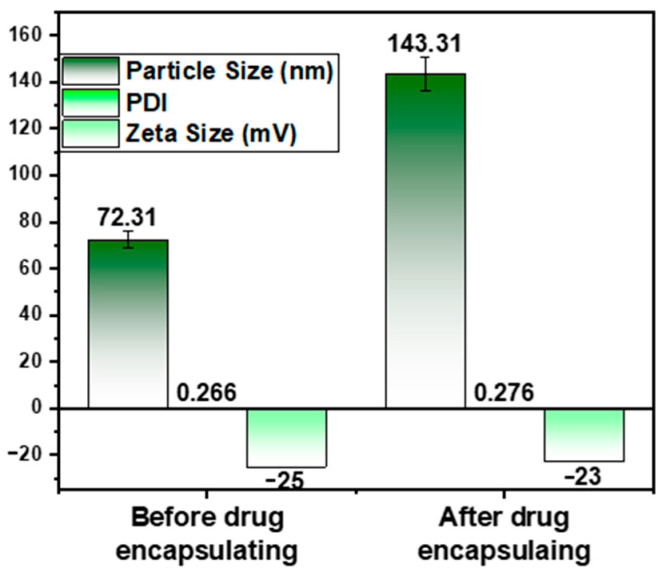
Graphical representation of particle size, PDI, and zeta potential of isoeugenol liposomes before and after encapsulation.

DLS technology was used to measure particle size, PDI, and zeta potential before and after isoeugenol encapsulated in liposomes, as shown in Figure 4. From Figure 4, it is clear that the particle size was found to be 72.31 ± 3.615 nm and after encapsulation of isoeugenol, the particle size was found to be 143.31 ± 7.165 nm. There is a marked difference in the before and after encapsulation of isoeugenol into the liposomes. Some essential oils can be safely encapsulated in a liposome with particles anywhere between 50 nm to 300 nm in size [35]. PDI is a measure of particle size distribution that can show that the prepared isoeugenol liposomes have uniform stability. The PDI was observed to be 0.266 ± 0.0133 before and 0.276 ± 0.0125 after the encapsulation of EOs. The PDI was observed to be within the range. A PDI of less than 0.1 indicates that a population is homogeneous, while a PDI greater than 0.3 indicates that a population has a significant degree of heterogeneity [36]. The zeta potential of isoeugenol liposomes was observed to be −25 ± −1.25 mV before encapsulating the drug, and after encapsulating isoeugenol, the zeta potential was found to be −23 ± −1.15 mV. As per the literature, a minimum potential of 30 mV is required to obtain a physically stable formulation [37]. On the basis of this, it can be concluded that the developed isoeugenol liposomes were in the nano range with good PDI and zeta potential. This indicates stable formulation.

### 2.5. Morphology

**Figure 5:**
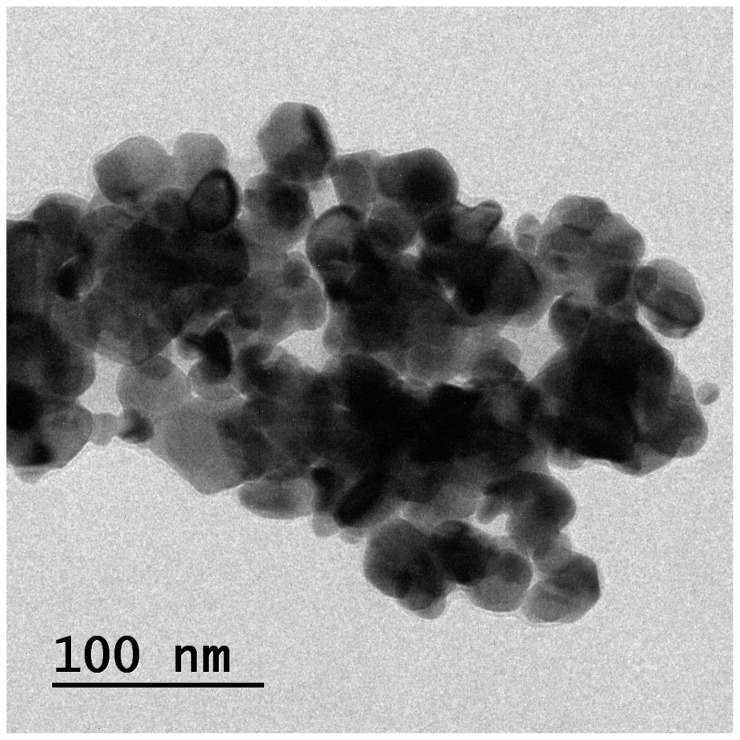
Morphology of isoeugenol loaded liposomes by TEM technique.

The morphology of the isoeugenol-encapsulated liposomes was observed under TEM, as shown in Figure 5. Isoeugenol-encapsulated liposomes were presented as spherical structures with smooth surfaces and regular shapes. From the image, it is clear that there is an encapsulation of the EOs. The image also supports and shows that the interaction between liposomes and essential oil significantly affects the structure of the formulation. The particle size was 100 nm, less than what was observed under DLS. It is mainly because DLS measures the hydraulic radius, and many factors affect it and use electrostatic resistance to stabilize the system [38]. From the figure, it can be observed some particle aggregation, which may be attributed to sampling methods or wall material properties [39].

### 2.6. Determination of Rheological Properties

Rheological behavior is essential since the dosage administered should distribute uniformly and stay on the skin. These paraments provide important information regarding the prepared liposomal gel’s appearance, homogeneity, viscosity, spreadibility, and pH. The isoeugenol-loaded liposomes were transparent and excellent in appearance, with no visible aggregates. The gel was homogenous throughout the system. The viscosity of the formulation was observed to be 38,791 cps. The viscosity reading is within the range of 35,000 to 40,000 cps of current applications. The research in rheology revealed the characteristics of thinning of rotary shear (pseudoplastic flow) in developed topical gels [40]. The pH of the prepared liposomal gel was observed to be 6.4. The pH was observed to be acceptable for topical drug delivery. The spreadability was observed to be 8.14 cm. Hence, from the rheological data, it can be concluded that the developed isoeugenol-loaded liposomes are homogenous with good spreadability and viscosity. Hence, it can also avoid the liposomes from the drug-leakage issue.

### 2.7. In Vitro Cell Viability Assay

**Figure 6:**
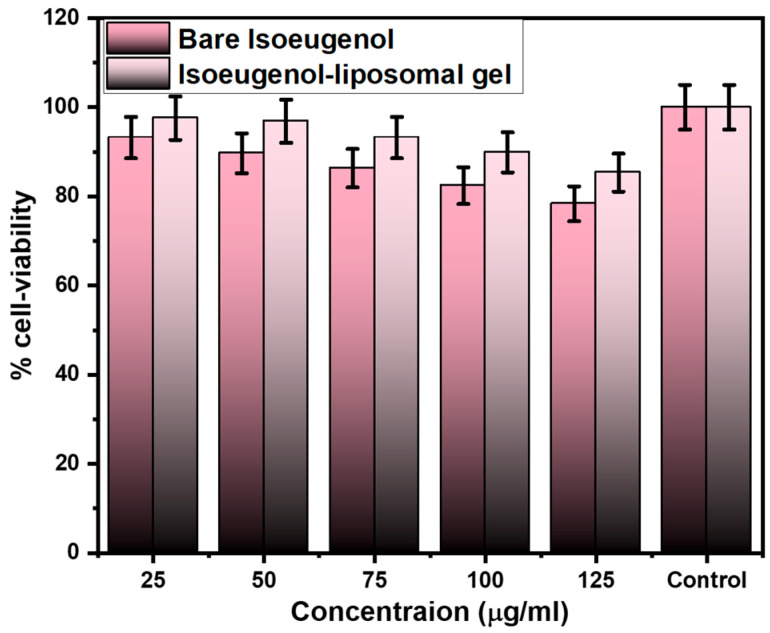
In vitro cell viability assay of bare isoeugenol and isoeugenol-liposomal gel against HaCaT.

The MTT assay is conducted to evaluate the cell viability, namely the human keratinocyte line (HaCaT) cells for both bare isoeugenol and isoeugenol-liposomal gel in the concentration ranging from 25 µg/mL to 125 µg/mL, as shown in Figure 6. The graph shows that the bare isoeugenol and isoeugenol-liposomal gel were safe for human use, with more than 75% of the cell-viability study. At a 25 µg/mL concentration, the cell viability was reported to be 93.19 ± 4.65% (bare) and 97.56 ± 4.87% (isoeugenol-liposomal gel). At a maximum, 125 µg/mL concentration, the cell viability was 78.34 ± 3.91% (bare) and 85.34 ± 4.26% (isoeugenol-liposomal gel). This data shows that the cell was viable, and no toxicities were observed. Hence, it can be concluded that bare isoeugenol and isoeugenol-liposomal gels were safe for human use.

### 2.8. Drug Release and Kinetic Studies

**Figure 7:**
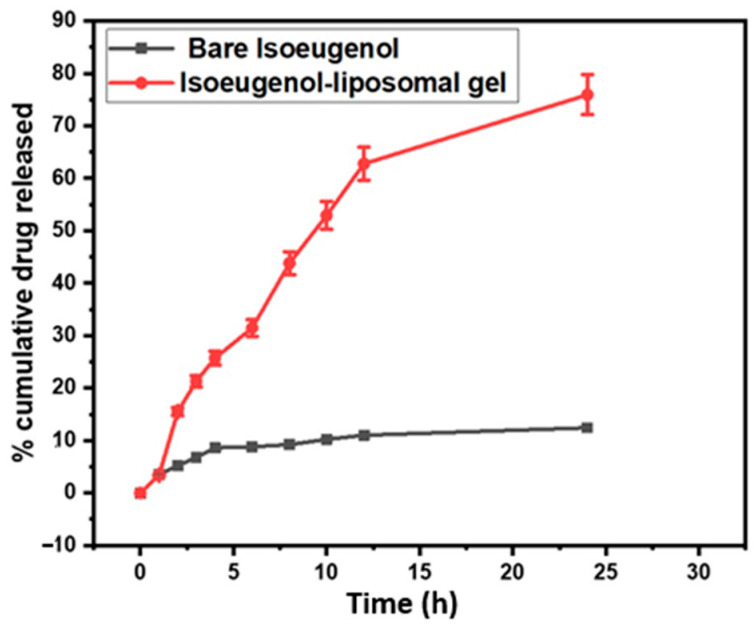
Comparative in vitro drug released; a study of bare isoeugenol and isoeugenol-liposomal gel in pH 6.0 media for 24 h. Study is performed in triplicates and all data are expressed as the mean ± standard deviation.

The in vitro drug releases to study for both bare isoeugenol and the isoeugenol-liposomal gels were conducted in a pH 6.0 buffer solution for 24 h, as shown in Figure 7. The sample was withdrawn from the release media, further diluted with the media buffer, and then evaluated immediately with the UV technique. From the graph, it is clear that the drug released was observed to be minimal, i.e., 6.85 ± 0.342% (3 h), 11.02 ± 0.551% (12 h) and 12.45 ± 0.622% (24 h). Whereas in the case of isoeugenol-liposomal gel, the drug released was observed to be very high, i.e., 21.32 ± 1.066% (3 h), 62.78 ± 3.13% (12 h) and 75.95 ± 3.79% (24 h). The outcomes of the results showed that the encapsulation of the drug increases the solubility of the drug, and hence in vitro drug release was observed to be more than bare.

The data obtained from the drug-released study was fitted into kinetic modeling and compared as given in Table 1. From the graph, it is clear that gel followed first-order release kinetic with r^2^ of 0.9782. This means that the drug release rate depends on the drug’s concentration. Additionally, encapsulating the isoeugenol into a liposomal gel carrier increases the solubility at skin pH.

### 2.9. Minimum Inhibitory Concentration (MIC)

MRSA bacterial suspension (SA6538) was used to determine MIC values for bare isoeugenol, isoeugenol-liposomal gels, and blank liposomal gel, as given in Table 2. The table showed that the MIC for bare isoeugenol was 44.67 µg/mL, whereas, in the case of isoeugenol-liposomal gels, the MIC was 8.236 µg/mL. This result indicates that encapsulating the isoeugenol in the liposomal increases the solubility and, further, antibacterial activity of formulation.

## 3. Conclusions

*S. aureus* bacteria are one of the most opportunistic pathogens that accumulate resistance to several classes of antibiotic treatment in healthcare settings. The abuse of unnecessary antibiotics has resulted in pathogens resistant to these drugs. MRSA is considered more dangerous among pathogen-resistant bacteria, with high morbidity and mortality rates in the community and hospitals. To solve the resistance against MRSA, the selected essential oils as an alternative therapy due to their reported pharmacological properties. The essential oil of isoeugenol is an effective antibacterial agent, but it is limited due to stability and hydrophobicity. So, to overcome this, the isoeugenol liposomes used a freeze–thaw technique by mainly using soya phosphatidylcholine and cholesterol in an equal concentration of 1:1. This technique was feasible and effective in preparing liposomes. The prepared liposomes were evaluated for different physio-chemical characterization. After characterization, it was incorporated into 0.5% of Carbopol 940 gel. The prepared isoeugenol-liposomal gel was safe for human use with increased anti-microbial properties. Using molecular docking and dynamics simulation techniques, this current study explains the intermolecular interaction of isoeugenol with penicillin-binding protein 2a, which is expected to assist lead optimization and design better antibacterial agents.

## 4. Materials and Methods

Isoeugenol, soya phosphatidylcholine, cholesterol, Carbopol 940, and triethanolamine were purchased from Sigma-Aldrich, (St. Louis, MO, USA). All the reagents used in this study were analytically graded. Methicillin-resistant *Staphylococcus aureus* (SA6538) was obtained from the Department of Pharmaceutics, College of Pharmacy, King Saud University, Riyadh. The other chemicals used in this present study were also procured from Sigma Merck. Nutrient media and Sabouraud dextrose were purchased from HiMedia (Mumbai, India).

### 4.1. Molecular Docking Studies

#### 4.1.1. Protein Preparation

The three-dimensional crystal structure of the penicillin-binding protein 2a from methicillin-resistant Staphylococcus aureus (MRSA) elucidated by X-ray diffraction (PDB ID:4CJN) having a resolution of 1.95 Å was retrieved from the Research Collaboratory for Structural Bioinformatics Protein Data Bank (RCSB PDB, http://www.rcsb.org/pdb/home/home.do, (accessed on 5 November 2022). The protein was examined for anomalies such as missing residues or atoms in Biovia Discovery Studio Visualizer 2021. Gasteiger charges were added to each atom in MGL Tools 1.5.7 and saved in pdbqt format as receptor file to be used in the next step [41].

#### 4.1.2. Ligand Preparation

Chemical structure of isoeugenol (CID: 853433) was obtained from PubChem database in sdf format and converted to pdb format in Biovia Discovery Studio Visualizer 2021. The ligand was handled in MGLTools 1.5.7 for merging all non-polar hydrogens and defining the number of rotatable bonds as well as torsion tree. Gasteiger method was used for adding partial atomic charges and finally saved in pdbqt format. The prepared ligand was enabled for flexible molecular docking in the active site of penicillin-binding protein 2a in the next step.

#### 4.1.3. Molecular Docking Study

Molecular docking study was performed with AutoDock Vina 1.1.2, implementing the default optimization parameters [42]. The native co-crystallized ligand (E)-3-(3-carboxyphenyl)-2-(4-cyanostyryl)quinazolin-4(3H)-one was used to describe the docking area encompassing a grid of 1 Å spacing having dimensions of 18, 18, and 18 points in x, y, and z directions, respectively. The grid-box was placed at allosteric binding site of PBP2a. Upon successful completion of docking, top 10 molecular conformations were processed to analyze their corresponding affinity in terms of binding energy and visualized in Biovia Discovery Studio Visualizer 2020, Ligplot plus, and Pymol 2.5 programs in order to understand the intermolecular interactions profile.

### 4.2. Molecular Dynamics Simulation

Using Desmond 6.1 molecular dynamics (MD) simulation computations, the thermodynamic behavior and stability of the best-ranked conformation of isoeugenol in a complex with penicillin-binding protein 2a were studied [43,44]. The isoeugenol-protein complex was placed inside an orthorhombic box with 10 buffer regions between protein atoms and box sides and filled with 30,294 molecules of water. For the MD calculations, the SPC model and the OPLS3e force field were used [45]. The system was neutralized with 88 Cl and 84 Na+ ions, at a constant salt concentration of 0.15 M, which reflects the physiological concentration of monovalent ions. The NPT ensemble was used, with the temperature and pressure set to 300 K and 1.01325 bar, respectively. A simulation time of 250 ns was set, with trajectories recorded every 250 ps. For short-range van der Waals and Coulomb interactions, a cut-off radius of 9.0 was utilized. The Nose–Hoover thermostat [46] and Martyna–Tobias–Klein [47] techniques were used to keep the system temperature and pressure stable. The integrator of the reference system propagator algorithm (RESPA) was used to integrate the equations of motion with an inner time step of 2.0 fs for both bonded and non-bonded interactions within the short-range cut-off [48]. For precise and efficient assessment of electrostatic interactions, the particle mesh Ewald approach was applied [49]. The system was reduced and equilibrated with Desmond’s default protocols. The Desmond package’s simulation interaction diagram protocol was used to examine the trajectory files upon completing the simulation.

### 4.3. Preparation of Isoeugenol-Loaded Liposomes

The freeze–thaw technique was used to prepare isoeugenol-loaded liposomes. Mainly soy phosphatidylcholine and cholesterol in an equal concentration of 1:1 were used. It was then dissolved in chloroform and methanol (100 mg/mL) [50]. A 1:1 ratio of soya lecithin to cholesterol formed more uniform liposomes with desirable characteristics, such as high encapsulation efficiency, according to our study. After this, the solution was dried under the vacuum at 50 °C using a rotary evaporator at 200 rpm. After the drying process, the round bottom flask was removed and allowed to be kept in a vacuum desiccator at room temperature overnight. The pH 7.4 phosphate buffer was added to this. To hydrate the lipids, the mixture was heated for an hour at 60 °C while being stirred constantly at 400 rpm. To get a suspension of liposomes, the medium was homogenized in a bath sonicator at 60 °C for 20 min. Following this, encapsulated isoeugenol in liposomes using 10% of essential oil in a buffer with a pH of 7.4 and kept it in the vortex for 10 min for uniform mixing. After being frozen in ice ethanol or acetone for 5–10 min, the solution was allowed to thaw at room temperature. The freeze–thaw cycle was repeated thrice, and the samples were centrifuged at 10,000 rpm for 30 min [51].

### 4.4. % Entrapment Efficiency (% EE)

The % EE of the liposome loaded with isoeugenol was determined by measuring the concentration of free drugs in the diffusion medium. In addition, about 5 mL of an undiluted sample of produced liposomes of isoeugenol dispersion was obtained and placed in the centrifuge tube. It was centrifuged for half an hour at 16,000 rpm. After that, a Whatman filter paper with 0.22 mm-sized pores was used to filter the supernatant. The aqueous phase’s free isoeugenol was determined by UV-Vis spectrophotometry at absorption maximum of isoeugenol is around 284 nm after appropriate dilution. A standard concentration range for isoeugenol was 0.1–10 mg/mL with correlation coefficient (r^2^) 0.998.

For each drug, the % EE was calculated using the equation below and shown as mean values ± SD [52]:

$$\%\mathrm{EE}=\frac{\left(Total\;amount\;of\mathrm{isoeugenol}\;added-Amount\;of\;free\;\mathrm{isoeugenol}\;in\;supernatent\right)}{Total\;amount\;of\;\mathrm{isoeugenol}\;added}\times 100\ldots$$

### 4.5. Determination of Particle Size, Size Distribution and Zeta Potential

Dynamic light scattering (DLS) was used to measure the mean particle size (z-mean) and particle distribution index (PDI) of isoeugenol-loaded liposomes with an Anton Paar, Austria. Measurements were made at a temperature of 25 °C and an angle of 173° to limit the effects of multiple scattering, which occurs when light scattered by 1 particle is scattered by other particles. Before measuring, all samples were diluted with distilled water to obtain an appropriate diffusion intensity. ZETA potentials were determined at 25 °C with the zeta potential dynamic light diffusion [53]. All samples were balanced for 120 s and then tested for 20 cycles. The results were given as the average of the measurements from the three.

### 4.6. Morphology

To prepare the samples for examination with a transmission electron microscope (TEM), 1 mg of the freeze-dried product was first dispersed in 600 L of Milli-Q water, and then 1 drop of this solution was placed on a copper grid. Subsequently, the analysis was performed. The sample was dried by air and then examined with the help of an electron transmission microscope (JEM-100CXII, Hitachi Co., Ltd., Tokyo, Japan).

### 4.7. Preparation of Isoeugenol-Loaded Liposomes Gel

First, 0.5 % *w*/*v* Carbopol 940 dispersion was made by dissolving it in distilled water (100 mL) and constantly stirring it with a paddle stirrer. It was allowed to hydrate for twelve hours. Glycerol (10% *w*/*w*) was added to the dispersion. To maintain the pH of 6, triethanolamine was added slowly to the carbomer gel under the complete stirring condition at 1200 rpm. The obtained dispersion gel was homogeneous. The carbomer gel was then shaken with a probe at an amplitude of 20 MHz for 10 s, followed by 1 min of vortexing. After this, isoeugenol-loaded liposome suspension and carbomer gel dispersion in the ratio of 3:1 was mixed by vortexing and stirring for 5 min. After mixing, the isoeugenol liposomal gels were kept overnight at room temperature [54]. The composition of gel is summarized in Table 3.

### 4.8. Determination of Rheological Properties

To determine the rheological characteristics of the isoeugenol liposomal gel and keep it at ambient temperature before measuring it, we examined the following.

Viscosity: The apparent viscosity of the isoeugenol liposomal gel was determined using a Brookfield viscosity monitor. For this purpose, the viscometer was operated at low rotational speeds, i.e., 0–3–6 rpm. Low RPMs help to preserve the structure of the liposomal gels. Since this was a purely digital instrument, the viscometer could read the viscosity. 

Spreadability: Spreading the gel helps to apply it evenly to the skin. To verify this, a spreadability study was conducted. The parallel plate method was used to evaluate and measure the spreadability of the liposomal isoeugenol gel. In this method, 0.5 g of isoeugenol liposome gel was taken and pressed between 2 horizontal plates (20 × 20 cm). On these horizontal plates, 500 gm weight was placed for 5 min. After this, the diameter of the circle was measured and denoted in cm.

pH: pH meter was used to measure the prepared liposomal gel’s pH. For this, the instrument was standardized to stabilize the pH using standard buffer solutions of pH 4.0 to 7.0. Once the pH meter was stabilized, the pH of the isoeugenol liposomal gel was measured. For this purpose, 0.5 g of gel was weighed, dissolved in 5.0 mL of distilled water, and the pH was measured.

### 4.9. In Vitro Cell Viability Assay

The cytotoxicity study was carried out to check whether the prepared formulation is safe for human use or not in human keratinocyte line (HaCaT) cells by modification in the existing technique published previously [55,56]. The HaCaT cell lines were obtained from King Saud University, Saudi Arabia. For this purpose, 200 µL of cell suspension was added to a 96-well plate at the required cell density (20,000 cells per well) without the assay medium. Cells were grown in modified Dulbecco Eagle medium (DMEM) and supplemented with 10% (*v*/*v*) heat-activated fetal bovine serum. The cells were grown for approximately 24 h. An appropriate concentration of the test agent was then added.

Cell was constantly maintained at 37 °C in a humidified incubator with 5% CO_2_. Incubation was performed at 37 °C for 24 h in an atmosphere of 5% CO_2_. At the end of the incubation period, the plates were removed from the incubator. The spent media was removed, and the MTT reagents were added to a final concentration of 0.5 mg/mL of the total volume. The plates were covered with aluminum foil to avoid exposure to light. The plates were placed in an incubator for 3 h. The MTT reagents were removed, and 100 µL of DMSO solution was added. Gentle agitation in a gyro shaker improves dissolution. Sometimes, it may be necessary to move the tube up and down to completely dissolve the MTT formation crystals, especially in dense cultures. Absorbance is measured using a spectrophotometer or ELISA reader at a wavelength of 570 nm. The percent cell viability is calculated using the following formula:

$$\%\mathrm{Cell}\;\mathrm{viability}=\frac{\left(A570\;\mathrm{nm}\;\mathrm{treated}\;\mathrm{cells}\right)}{\left(A570\;\mathrm{nm}\;\mathrm{untreated}\;\mathrm{cells}\right)}\times 100$$

### 4.10. Drug Release and Kinetic Studies

A vertical diffusion model was used to perform the in vitro drug release study of isoeugenol liposome gel. For this purpose, the PBS buffer medium with a pH of 6.0 at 32 °C was chosen. In this medium, there were two compartments, a donor compartment and a receptor compartment, separated by a semisynthetic cellulose acetate membrane. Then, 500 µL of the sample was added to the donor compartment. On the other hand, 50 mL of the receptor solution was added to the receptor compartment. Then, 2 mL of the sample was withdrawn regularly and replaced with PBS buffer pH 6.0 to maintain the sink condition. UV technique was used to analyze the solution [57]. The in vitro drug release study was in triplicate, and the values were reported as mean ± SD. After obtaining the data from the in vitro drug release study, the mathematical models to describe the release kinetics of the liposomal isoeugenol gel were carried out.

### 4.11. Minimum Inhibitory Concentration (MIC)

#### Preparation of Inoculum

To perform the MIC study, the sterile microtiter plate technique was used. In this procedure, the inoculum is first prepared. For this purpose, the stock solution of pure isoeugenol and isoeugenol liposome gel was prepared at a concentration of 1 mg/mL to ensure complete solubilization. Then, 100 µL of nutrient broth and Sabouraud dextrose broth were added to wells 1 to 10. Then, 100 µL of isoeugenol and isoeugenol liposome gel were added to the first and second wells. The solution was diluted sequentially in wells 1 to 10. To this, 100 µL of the MRSA bacterial suspension (SA6538) from wells 1 to 10 was added. In well 11, 100 µL of the bacterial suspension and 100 µL of sterile broth were introduced to serve as a positive control or growth control. In well 12, 200 µL of sterile nutritional broth and Sabouraud dextrose broth was taken as a negative or sterility control, respectively. The plates were kept for incubation by maintaining the temperature at 37 °C overnight. After the incubation, the absorbance of the sample in each well was measured with an ELISA reader (Erba) at a wavelength of 640 nm. The concentration of the sample and standard that inhibits 50% of bacterial growth was calculated for each bacterium. This study was performed in triplicate, and data were presented in mean ± SD.

## Figures and Tables

**Table 1:** r^2^ value of bare isoeugenol and isoeugenol-liposomal gel using different kinetic studies for 24 h. Date presented is taken from in vitro drug release study.

Kinetic Model	Bare Isoeugenol	Isoeugenol-Liposomal Gel
Zero-order	0.7887	0.9643
First order	0.7969	0.9782
Higuchi	0.955	0.9274
Kors–Peppas	0.8663	0.9758
Hixson	0.7933	0.9756

**Table 2:** MIC of the bare isoeugenol, isoeugenol-liposomal gel, and blank gel against MRSA. Study is performed in triplicates, and all data are expressed as the mean ± standard deviation.

Treatment Given	MIC (µg/mL)
Bare isoeugenol	44.67 ± 0.91
Isoeugenol-liposomal gels	8.236 ± 0.67
Blank liposomal gel	N.A.

**Table 3:** The composition of the gel formulation.

Ingredients	Concentration/Amount
Carbopol 940	0.5 % *w*/*v*
Distilled water	100 mL
Glycerol	10% *w*/*w*
Triethanolamine	(amount added to maintain pH 6)
Isoeugenol-loaded liposome suspension and carbomer gel dispersion	3:1

## Data Availability

The data presented in this study are available on request from the corresponding author.
